# Roles and challenges of construction firms and public health entomologists in ending indoor malaria transmission in African setting

**DOI:** 10.1186/s12936-016-1607-9

**Published:** 2016-11-14

**Authors:** Eliningaya J. Kweka

**Affiliations:** 1 Division of Livestock and Human Diseases Vector Control, Tropical Pesticides Research Institute, P.O. Box 3024, Arusha, Tanzania; 2 Department of Medical Parasitology and Entomology, Catholic University of Health and Allied Sciences, P.O. Box 1464, Mwanza, Tanzania

**Keywords:** Malaria, Indoor transmission, House modification, Entomologists, Construction firms

## Abstract

Indoor malaria transmission reduction across sub-Saharan Africa has been attained through implementation of long-lasting insecticidal nets and indoor residual spray interventions with small-scale larval source management. Improvement of house structures in sub-Saharan Africa can lead to zero indoor malaria transmission with evidence from West Africa, East Africa and Middle East countries. Residual malaria transmission cannot be targeted well with LLINs and IRS alone, but with incorporation of house structures modifications it may be possible.

## Background

In recent past, malaria control has been successful for reduction of mortality and morbidity related cases upto 438,000 malaria deaths (range 236,000–635,000) worldwide per annum [1], which is a decrease of malaria mortality cases by 60% since 2000 [2]. The main routes which so far have played a major role in reduction of the observed malaria mortality and morbidity includes (1) proper diagnosis and the use of appropriate anti-malarial drugs; (2) the use of adult vector control tools such as indoor residual spray (IRS) and long-lasting insecticidal nets (LLINs); and (3) larval source management in some rural and urban areas [1]. For past two decades, insecticide resistant among malaria vectors has been reported for different classes of insecticides used for vector control [3]. Eighty percent of malaria cases have been found to be transmitted indoors in sub Saharan Africa [4]. The use of LLINs and IRS indoors has reduced but not limited the vector house entry and feeding behaviour which has cause to have a substantive changes in vector composition in many areas with *Anopheles gambiae s.s.* all but disappearing, leaving *Anopheles arabiensis,* which is known to be capable of feeding extensively on humans early in the evenings, before human go indoors, as the only remaining vector species of the *An gambiae s.l.* complex [4]. The use of LLINs and IRS indoors has reduced vectors house entry behaviour and caused species shifts due to indoor insecticidal pressure [5, 6], shifts to early-evening or early-morning biting [7]; shifts to exophagy [6, 8]; shifts to zoophily [9] and shifts to exophily [10]. More efforts are needed to address restriction of vector house entry behaviour and zero indoor malaria transmission. This commentary work put more emphasis on the way African house improvements could lead to the indoor vector abundance reduction and zero indoor malaria transmission and restrict to have the malaria foci.

The main routes for mosquito house entry are eaves, open window and doors [11–16]. The traditional African houses are short and have eaves, unscreened doors windows [17]. The short walls of the houses have enhanced mosquito to enter the house easily at the height of 2–2.5 m; windows are mostly located at the height 1–1.5 m above the earth surface. The behavioural response of mosquitoes to human or other host odour indoors has found that, mosquitoes to have ability of going through windows, open doors and eaves [18]. The house structure have changed in terms of height, sealed caves and with screened windows and door [12]. These changes have found the indoor vector density declining [12]. In other sites, malaria incidences found decreasing among household members with reference to house type, entry points blocking (windows and doors screening, and eaves blocking) [14, 19]. In Western Kenya, introduction of house sealing using locally available cheap resources found the indoor house vector density decreased and subsequently malaria reduction [20]. In the Sao tomé, Charlwood and others realized that, increasing the house height upholds vectors opportunity to enter the houses [21]. In the other trial conducted in West Africa by Njie and others found that, screening the houses most entry points eaves, doors and windows lead to reduced density of *An. gambiae s. l.* in doors but not for Culicine species [13]. Reduction of Anopheline house entry directly reduces the risk of indoor malaria transmission.

The major role to be played by the construction industry (private–public sector collaboration) and public health entomology include capacity building to human resource available in the firms already practicing construction on mosquitoes house entry control by house structure modifications which includes, house walls height increase, eaves sealing, and house window and door screening. Capacity building by introduction of modules for public health entomologists and structural engineering in colleges for adapting training curriculum to changing epidemiological and entomological determinants, more implementation or operational research.

Public health entomologists, who are part of National Malaria Control Programmes (NMCP’s) across sub-Saharan Africa, have to play the role of educating the community on vector behaviour and house entry points. The shortage of public health entomologists in sub-Saharan African might be a problem to reach the rural community [22], but the effect of house construction for vector control could be vivid when engineers and all construction firms will be fully integrated in public health. Integration of public health entomologists and engineering firms can give better solution in vectors house entry control with use of existing evidences [11, 21]. Public health and field entomologists have declared that the IRS and LLIN lost efficacy against mosquitoes due to the insecticide resistance developed in some vectors [23]. The most reliable method for the fight against indoor vector density control could only be modification of the house structures (e.g., by sealing of eaves, screening of doors and windows). This could be the best supplement of larval source reduction, IRS and LLINs coverage. The larval source reduction in combination with IRS and LLINs have been great in vector and disease transmission reduction [24], but still there is residual malaria transmission in several part of Africa [25].

In Africa, the major challenges expected towards house modification for malaria control are traditions (for some tribes on house style) socio-economic status [26] and settlement problem for nomadic communities [27] and refugees. In Africa, some nomadic tribes are still hold on to their traditions which include none permanent low quality house style and poor access to health system [28]. These are among tribes that do not change their traditional life style easily and hence challenges to houses improvement still need more effort. These challenges can be resolved with the use of traditional leaders of each community who can champion the house style improvements in their communities in collaboration with public health entomologists. Traditional leaders are used as informers and changers in community in Africa [29]. Social economic status of African rural communities still a challenge on attaining better health and live hood, mostly in house infrastructure [30]. The main source of income in rural areas of Africa is agriculture which depends on natural rain cycles with small scale irrigation [30]. Unreliable rains have reduced harvests and subsequently family income due to climate changes and unpredicted climate changes [31]. The outcomes of low-income has been observed and indicated by the extreme poverty. The improvement of houses for malaria control shall be hindered on this circumstance of poverty. Nomadic communities have no settled home, move from place to place for fruit gathering, hunting, finding pasture for livestock, or otherwise making a living [32]. These communities have not permanent house structures as they migrate from time to time to meet their daily needs. More efforts are needed to motivate this community for permanent settlements by government and political leaders of their communities. Refugees in sub-Saharan Africa are the products of internal political conflict in different countries, such as Eritrea and southern Sudan, which makes Ethiopia a most refugee hub of Africa [33] followed by Kakuma camps in Kenya [33]. These camps have been having high malaria incidences due to poor sheltering and health services [34]. These camps are temporary for humanitarian basis and have to complement the standard practices for malaria control in humanitarian emergencies to increase indoor infectious mosquito bites protection and supplement protective gears for outdoor protections such as repellents [35]. More efforts to avoid having malaria foci for stabilizing political status in African countries are needed with the management of residual malaria transmission. Deployments of tools such as insecticide-treated plastic sheeting and treated blankets [36], Zero Fly [37], non-mesh LLINs products [38] and use of repellents both synthetic and plant based [39] for malaria control in conflict and emergency humanitarian situations. Government and private firms should ensure to promote these tools in terms of safety, acceptability and their availability upon demand. Strategise on purchase and distribution to enhance manufacturer confidence for production to meet the community demand. The use of non-pyrethroid compounds should be also promoted to manage the growing pyrethroid resistance such as non-pyrethroid treated wall lining materials [40].

## What is the way forward?

Malaria control in sub-Saharan Africa should be taken as a multi-sectorial effort to maintain and exceed attained control efforts [41]. There should be a policy in place for integrating construction firms and NMCP’s programmes in Africa for designing better houses that bring indoor malaria transmission to zero. Refugees and nomadic life styles should be discouraged by head of African states with alternatives sources of better income for permanent settlements. Majority (80%) of the African population in rural areas rely on rain based agriculture for food and cash crop productions [42]. Climate change has lead to reduced and unreliable rains, hence governments should address better infrastructure for irrigation to enhance better family income, housing and increased social economic status (improved livelihood).

## Conclusions

This commentary demonstrates that collaboration between public and private sectors under the national malaria control programmes to assess options for addressing residual transmission can have measurable outputs. This can be achieved under programmatic conditions through pilot studies with strong monitoring, evaluation and operational research components, similar to what has been done by the Onchocerciasis Control Programme in West Africa.

## Abbreviations

LLIN: long-lasting insecticidal nets
IRS: indoor residual spray
NMCP: National malaria control programmes
